# *Cassia fistula* Leaves; UHPLC-QTOF-MS/MS Based Metabolite Profiling and Molecular Docking Insights to Explore Bioactives Role towards Inhibition of Pancreatic Lipase

**DOI:** 10.3390/plants10071334

**Published:** 2021-06-29

**Authors:** Zain Ul Aabideen, Muhammad Waseem Mumtaz, Muhammad Tayyab Akhtar, Muhammad Asam Raza, Hamid Mukhtar, Ahmad Irfan, Syed Ali Raza, Tooba Touqeer, Muhammad Nadeem, Nazamid Saari

**Affiliations:** 1 Department of Chemistry, University of Gujrat, Gujrat 50700, Pakistan; chzain321@yahoo.com (Z.U.A.); muhammad.waseem@uog.edu.pk (M.W.M.); asamgcu@yahoo.com (M.A.R.); tuba.toqir@gmail.com (T.T.); nadeembajk@gmail.com (M.N.); 2 Institute of Industrial Biotechnology, GC University Lahore, Lahore 54000, Pakistan; hamidmukhtar@gcu.edu.pk; 3 Department of Chemistry, Faculty of Science, King Khalid University, P.O. Box 9004, Abha 61413, Saudi Arabia; irfaahmad@gmail.com; 4 Research Center for Advanced Materials Science (RCAMS), King Khalid University, P.O. Box 9004, Abha 61413, Saudi Arabia; 5 Department of Chemistry, GC University Lahore, Lahore 54000, Pakistan; chemstone@yahoo.com; 6 Department of Food Science, University Putra Malaysia, Serdang 43400, Malaysia

**Keywords:** ultrasonicated extraction, antioxidant, antiobesity, molecular docking, *C. fistula*

## Abstract

The present work was aimed at investigating hydroethanolic leaf extracts of *Cassia fistula* for their antioxidant and pancreatic lipase (PL) enzyme inhibitory properties. The most active extract was selected to profile the phytoconstituents by UHPLC-QTOF-MS/MS technique. Among the tested extracts, the 80% hydroethanolic extract exhibited the maximum levels of total phenolic and flavonoid contents (TPC and TFC) with a contribution of 201.3 ± 2.6 mg of gallic acid equivalent per gram of extract (GAE/g extract), and 116.3 ± 2.4 mg of rutin equivalent per gram of extract (RE/g extract), respectively. The same extract also showed promising 2,2-diphenyl-1-picrylhydrazyl (DPPH) radical scavenging and PL inhibitory activity with an IC_50_ (half maximal inhibitory concentration) of 30.5 ± 2.8 µg/mL and 17.31 ± 1.18 μg/mL, respectively. The phytochemical profiling of 80% hydroethanolic extract confirmed the presence of 23 metabolites of immense medicinal significance. Docking studies were conducted to investigate the potential interactions of compounds identified in the study. The docking study-based binding energy data and the interaction scheme both revealed the possible role of the identified compounds towards PL inhibitor. Moreover, energies of frontier molecular orbitals (FMOs), ionization potentials (IP), electron affinities (EA) and molecular electrostatic potentials (MEP) were also explored. The findings of the current work suggest that *C. fistula* is a promising natural source of antioxidant and antiobesity agents, which may be exploited to add pharmacological functionalities to food.

## 1. Introduction

Obesity is one of the leading global health problems that enhances risks of chronic diseases such as diabetes, heart diseases, metabolic syndrome, strokes, hypertension, mental disorders and cancer [1,2]. The prevalence of obesity is very widespread, and about 500 million people of the adult population are reported as obese worldwide [3]. Obesity generally results from an imbalance between high caloric diet intake and expenditure of energy. The extra energy is stored in adipose tissue or adipose mass. PL is important among the enzymes that catalyze the hydrolysis of triacylglycerols producing diacylglycerols, monoacylglycerols and free fatty acids, which can easily be absorbed into the body. Inhibition of PL is adopted as a rational approach to control obesity due to fact that up to 70% of total dietary fat hydrolysis is achieved by this enzyme [4]. The mechanism involved is the inhibition of absorption of dietary triglycerides, as triglycerides are the major contributor towards excessive calories which disturb normal metabolic function. Inhibition of PL does not change or modify central mechanisms of the body that impart safety to this therapeutic approach, making it a valuable tool for the treatment of obesity [2,5]. Powerful synthetic drugs (including orlistat, phentermine-topiramate, lorcaserin, and liraglutide) are available in the market for the management/treatment of obesity. However, these drugs are not only very expensive, but have serious side complications including headache, insomnia, agitation, palpitation, nervousness and heart attack [6]. Therefore, there is great need to investigate cheap and safe natural products (particularly from medicinal plants) with promising antilipase activity. Polyphenolic compounds can influence the functions of enzymes that control protein, glucose and lipid metabolism. Various researchers have reported that extracts of grape seed [7], berries rich in ellagitannins [8], lotus containing flavonoids [9] and green, black and white tea [10], can inhibit lipase activity in vitro. Epidemiological investigations have indicated that oxidative stress plays an obvious role in induction of obesity and related complications [11,12]. Oxidative stress triggers obesity by augmenting white adipose tissue mass and influencing the intake of food. Studies have also shown that oxidative stress can enhance differentiation of adipocytes, proliferation of preadipocytes and size of mature adipocytes. Reactive oxygen species participate in the management of body weight by exerting adverse effects on hypothalamic neurons that control hunger and satiety behavior [12,13]. Synthetic antioxidants like butylated hydroxyanisole (BHA) and butylated hydroxytoluene (BHT) are used to treat oxidative stress, but the serious health deteriorating effects associated with these compounds provide impetus to explore natural sources of antioxidants with no or tolerable toxicity [14]. Previous studies have indicated that medicinal plants can prevent or decrease oxidative deterioration of lipids, DNA and proteins, thus controlling neurodegenerative disorders [15]. Recently, researchers have focused their interest on plant-based functional foods/nutraceuticals and phytopharmaceuticals for management of obesity and its related complications, as they exert numerous beneficial effects with fewer or no side effects [16]. Most studies have shown that plant-based functional foods could normalize food intake, lipid metabolism, energy expenditure and adipocyte lifecycle by regulation of genetic or epigenetic mechanisms [17].

*Cassia fistula* Linn., a native to Pakistan, India, Myanmar and Sri Lanka, is a flowering plant of the family Fabaceae. It is widely known as *Cana fistula*, Amaltas, and Golden Shower [18,19]. *C. fistula* is a good source of naturally occurring biologically active secondary metabolites (anthraquinones, triterpenes, alkaloids, proanthocyanidins, catechins and flavonoids) which are used as dietary supplements, medicines and other valuable commercial products [20]. *C. fistula* is utilized to cure many ailments in traditional medicinal systems, including pruritus, leukoderma, haematemesis and obesity [21]. The antitussive, antipyretic, antimicrobial, antifungal, inflammation control and antioxidant potential of this plant have been reported [19,22]. The extraction process is a very important aspect to get maximum pharmacological output from a particular plant part. Recent advances in extraction techniques have revitalized the natural product field with respect to improved extraction and resulting better medicinal activities. Microwave-assisted extraction, super critical fluid extraction and ultrasonicated-assisted extraction for qualitative and quantitative analysis of polyphenolics in combination with techniques including GC-MS and LC-MS, GC-TOF-MS, UPLC-MS, and UPLC-QTOF-MS, have become valuable tools in the metabolic profiling of plant samples [14,23,24].

Considering the well-reported medicinal usages of *C. fistula*, the present work was performed to explore the antioxidant and antiobesity effects of hydroethanolic *C. fistula* leaf extracts, which, in turn, might help the future formulation of antiobesity functional foods/phytopharmaceuticals with least/no adverse side effects. Identification of predominant compounds present in the most potent *C. fistula* leaf extract was performed via a sensitive and reliable technique i.e., UHPLC-QTOF-MS/MS, followed by molecular docking and computational studies of identified bioactive compounds, to provide the scientific basis regarding antioxidant and antiobesity activity of *C. fistula*.

## 2. Results and Discussion

### 2.1. Extract Yield

Extraction is the separation of bioactive constituents from medicinal plants using specific solvents and adopting specific protocols. The aim of the extraction process is to take out plant metabolites from the complex plant matrix. The high demand for antioxidants stimulated ways to explore new methodological alternatives (cleaner and environment friendly) that could improve the yield of extracts and decrease the process cost [25,26].

**Figure 1:**
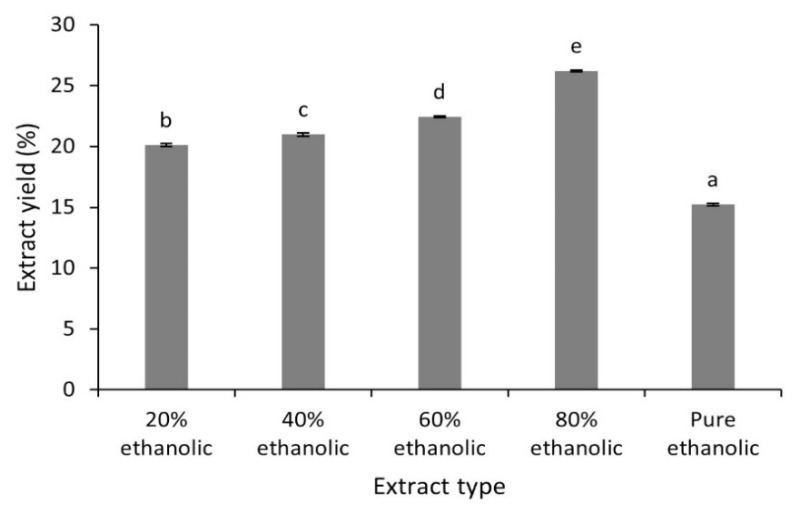
Extract yield (%) of different extracts from *C. fistula* leaves. Values that do not share a letter are statistically significant (*p* < 0.05).

The percentage yields of different hydroethanolic (20 to 80%) and pure ethanolic *C. fistula* leaf extracts are displayed in Figure 1. The extraction yields of these samples were in the range from 15.23 ± 0.09% to 26.21 ± 0.06%. The results revealed that the 80% hydroethanolic extract had the maximum extraction yield while the minimum extract yield was obtained by pure ethanolic extract. The solubility of phenolic compounds depends mostly upon the nature of solvents employed and their polarities. In the current work, results of ANOVA revealed that the influence of solvent polarity on extract yield was significant (*p* < 0.05). Mixtures of water and ethanol in different proportions can extract bioactive constituents from different plant parts. Although water has high ability to extract polar compounds due to its higher polarity compared to other solvents, it is unable to extract high carbon bond compounds, so solvents with suitable extraction coefficients may be prepared by mixing ethanol and water.

Previously reported extract yields of ethanol: water (9:1) and methanolic leaf extracts from *C. fistula* were 7% and 20.2%, respectively [27,28]. The values of extraction yields in the current work were different compared to those reported in literature. The slight variation could be attributable to the difference in polarities of the solvents used which also play an imperative role in increasing the solubility of phytocompounds [29].

### 2.2. Total Phenolic and Flavonoid Contents

Medicinal plants/herbs have gained considerable importance, being rich sources of therapeutic phytoconstituents that can lead to the development and formulation of novel functional foods/nutraceuticals or phytopharmaceuticals. Most of the plant-based bioactives, such as phenolics and flavonoids, have beneficial health effects. In medicinal plants, high contents of flavonoids and phenolics are associated with antioxidant activity, thus playing a significant role in prevention/treatment of several chronic disorders related to oxidative stress. Due to the beneficial phytocompounds in medicinal plants and trends in natural product consumption in nutraceuticals and the food processing industry, the exploration of medicinal plants is becoming increasingly important [25,30,31].

The amounts of TPCs and TFCs in the leaves of *C. fistula* are given in Table 1. The results show the difference in levels of TPCs and TFCs in all extracts. The TPCs varied from 138.5 ± 2.3 to 201.3 ± 2.6 mg GAE/g and the maximum amount was obtained with 80% hydroethanolic extract (201.3 ± 2.6 mg GAE/g), followed by 60% hydroethanolic extract (190.6 ± 3.0 mg GAE/g), 40% hydroethanolic extract (181.2 ± 1.9 mg GAE/g) and 20% hydroethanolic extract (152.19 ± 1.4 mg GAE/g). The lowest contents of phenolic compounds found in the pure ethanolic leaf extract might be explained by the lower solubility of polyphenols in this solvent system. A statistically significant difference of means (*p* < 0.05) was observed between the 80% hydroethanolic extract and other extracts. Likewise, the maximum amount of TFCs was seen in the 80% hydroethanolic extract with a value of 116.3 ± 2.4 mg RE/g, and it was different statistically, with *p* < 0.05, when compared with those of 60, 40 and 20% hydroethanolic and pure ethanolic extracts. In a previous study, the amount of TPCs and TFCs in aqueous extract of *C. fistula* were 43.57 ± 1.16 mg GAE/g extract and 4.99 ± 0.10 mg QE/g extract, respectively, which were less than the TPC and TFC values depicted in the current investigation [27]. The slight variation in amounts of TPCs and TFCs in the present work and those already reported might be due to various phytogeographic regions and plant nutrition [32].

### 2.3. DPPH Radical Scavenging Activity In Vitro

Oxidative stress-induced hypertension has promoted an increasing interest in therapeutic potential of antioxidants. The antioxidants could be utilized as supplements to manage different ailments resulting from the oxidative stress, including obesity. However, most of the studies have focused usage of synthetic antioxidants to reduce the harmful impacts of oxidative stress that are often not considered successful due to their myriad side effects [33]. Emerging evidence has suggested that natural products can attenuate the risk of oxidative stress and improve the immune system. Therefore, the exploration of natural products or medicinal herbs as promising therapeutic sources for the management of obesity has been promoted due to their minimal adverse effects [34]. In the current work, the antioxidant effect of *C. fistula* leaves was evaluated using DPPH radical scavenging assay.

**Figure 2:**
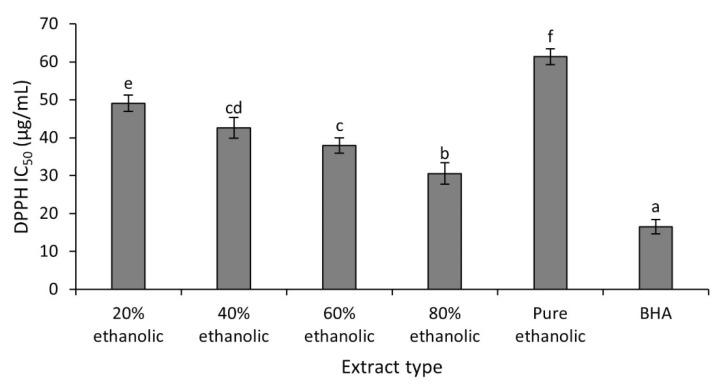
The DPPH radical scavenging activity of different extracts from *C. fistula* leaf. Values are noted with letters ^(a−f)^ to show statistically significant differences. Values sharing a letter are not significantly different (*p* > 0.05).

The DPPH radical scavenging assay is used frequently to determine antioxidant effects of plant extracts [11]. Figure 2 present the results (expressed in terms of IC_50_ values) of antioxidant activities obtained for the studied extracts and the positive control (BHA).

Evaluation of DPPH radical scavenging activity of various tested extracts indicated that the 80% hydroethanolic extract exhibited maximum activity with an IC_50_ of 30.5 ± 2.8 µg/mL, while minimum antioxidant activity was seen in the pure ethanolic extract with an IC_50_ of 61.3 ± 1.9 µg/mL. As revealed statistically, antioxidant potential of 80% hydroethanolic extract was maximum and different significantly (*p* < 0.05) compared to other extracts, but slightly less than that of BHA (IC_50_ of 16.55 ± 1.90 µg/mL). Results revealed that the 80% hydroethanolic leaf extract had significant antioxidant activity and might be able to protect the body from the damaging effects produced by oxidative stress. The antioxidant activity exhibited by a particular plant extract mainly depends upon the nature of the phytochemicals extracted from plant parts. However, their yields are affected by the solvent system used for extraction [11]. The difference in antioxidant effect of the current work and reported data could be linked to different level of polyphenolic compounds in the extracts due to solvent polarities and methods of extraction.

### 2.4. Antiobesity Activity of C. fistula

Obesity is a major public health concern worldwide and related to several chronic disorders. Several synthetic drugs are in use to cure obesity with variable results and adverse effects [6]. Inhibition of dietary absorption by inhibiting PL is one of the effective approaches for the management of obesity. Several medicinal plants have been reported as sources of PL inhibitors with limited adverse effects [2].

**Figure 3:**
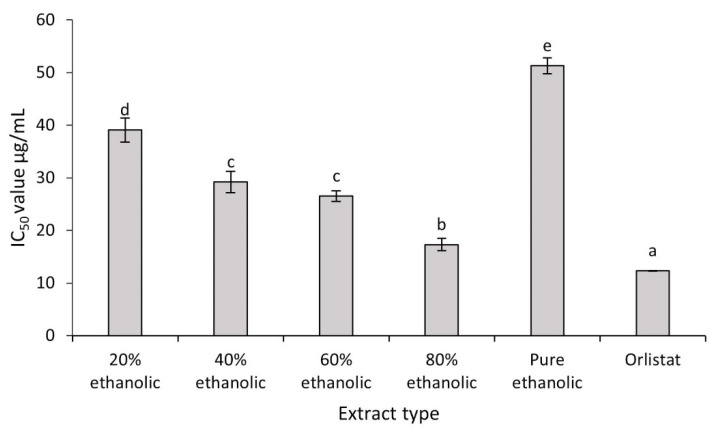
Inhibitory activities of different extracts of *C. fistula* towards PL. Values are noted with letters ^(a−e)^ to show the level of statistical significance (*p* < 0.05). Values sharing a letter are not significantly different (*p* > 0.05).

PL inhibitory effects were examined to determine the ability of *C. fistula* leaf extracts as antiobesity agents. Outcomes are presented in Figure 3.

All the extracts exhibited strong inhibition against PL, but inhibitory activity of the 80% hydroethanolic extract was maximum with an IC_50_ value of 17.31 ± 1.18 μg/mL. This value was quite impressive when compared with the standard PL inhibitor, orlistat, for which the IC_50_ value was 12.34 ± 0.02 μg/mL. These values were followed by 60% hydroethanolic (IC_50_ = 26.53 ± 0.99 μg/mL), 40% hydroethanolic (IC_50_ = 29.21 ± 2.03 μg/mL) and 20% hydroethanolic (IC_50_ = 39.11 ± 2.30 μg/mL) extracts. However, it was noticed that the IC_50_ values of both the 40% hydroethanolic and 60% hydroethanolic extracts were found statistically nonsignificant. Comparative analysis revealed that pure ethanolic extract exhibited the lowest enzyme inhibitory action among all extracts (IC_50_ of 51.3 ± 1.5 μg/mL). Findings were of great interest and revealed considerable inhibitory effect of *C. fistula* leaves towards PL and its potential for antiobesity functionality. A recent study linked the in vitro PL lipase inhibition by hydroethanolic extracts of *Taraxacum officinale* (*T. officinale*) with the blood biochemistry of obese mice treated with same extract. The *T. officinale* extract showed PL inhibition with an IC_50_ value 146.49 ± 4.24 µg/mL and its application on high fat diet-fed obese mice significantly improved the lipid profile by modulating their total cholesterol, high density lipoproteins and low density lipoproteins [35,36].

### 2.5. UHPLC-QTOF-MS/MS Based Metabolite Profiling

**Figure 4:**
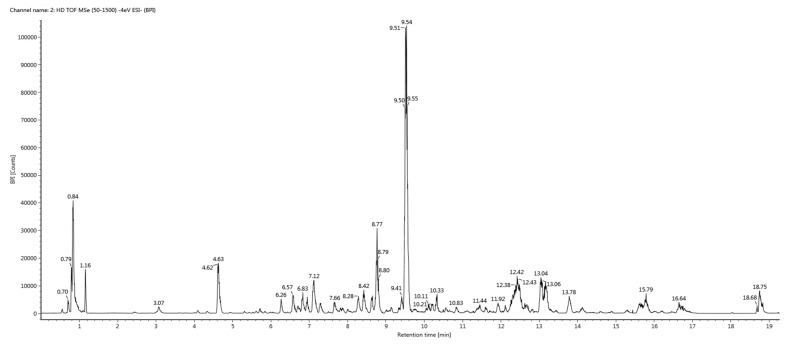
UHPLC chromatogram of 80% ethanolic extract of *C. fistula* leaf.

**Figure 5:**
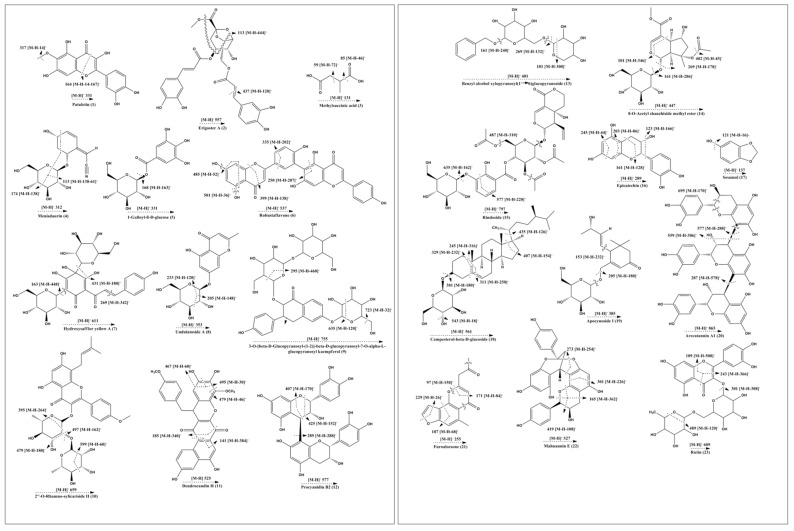
Possible fragmentation patterns of identified compounds.

Profiling of metabolites in the 80% hydroethanolic leaf extract (most potent) from *C. fistula* was conducted by employing UHPLC-QTOF-MS/MS. The base peak chromatogram of the tested sample is presented in Figure 4. Analytical information of all identified phytochemicals (including precursor ions ([M-H]^−^), predicted formula, mass error and MS/MS fragment ion peaks) is provided in Table 2, whereas most probable fragmentation patterns of all identified phytocompounds are displayed in Figure 5.

The UHPLC-QTOF-MS/MS analysis resulted in the identification of 23 metabolites in the 80% hydroethanolic leaf extract of *C. fistula*. Compound (**1**) appeared with an observed mass of 332.05 mDa, having the predicted formula C_16_H_12_O_8_ and three fragment ions at 317 [M-H-14]^−^, 300 [M-H-31]^−^ and 164 [M-H-167]^−^, suggesting possible elimination of CH_2_, CH_3_O and C_8_H_7_O_4_. On the basis of the proposed fragmentation pattern and reported literature data, compound (**1**) was identified probably as patuletin [37,38]. Compound (**2**) was identified as erigoster A, with predicted mass of 558.14 (C_27_H_26_O_13_) [39]. The precursor ion on fragmentation generated product ions at 437 [M-H-120]^−^ that might be obtained by the loss of C_7_H_4_O_2_ moiety, and 113 [M-H-444]^−^ due to possible loss of a C_22_H_20_O_10_ unit. Compound (**3**) was tentatively identified as methyl succinic acid with a predicted mass of 132.04 mDa (C_5_H_8_O_4_). In the mass spectrum of compound (**3**), the major fragment ions at 101, 85 and 59 appeared, which were characteristic fragmentation peaks for this substance [40]. Compound (**4**) was detected with an observed mass of 313.11 mDa (C_14_H_19_NO_7_). In the MS/MS spectrum, a prominent ion peak was produced at 174 [M-H-138]^−^, which might be due to loss of C_5_H_14_O_4_, which later produced another fragment at 113 [M-H-138–61]^−^ by possible removal of C_2_H_5_O_2_ from the ion at 174. Based upon mass spectral information, fragmentation pattern and an online data base (PubChem), compound (**4**) was identified probably as menisdaurin. The mass spectrum of metabolite (**5**) exhibited a molecular formula of C_13_H_16_O_10_ with a predicted mass of 332.07 mDa. The fragment at 168 suggested the possible loss of a glucose moiety [M-H-163]^−^ (C_6_H_11_O_5_). Metabolite (**5**) was thus tentatively identified as 1-galloyl-β-D-glucose, which agrees with literature data [41,42]. Compound (**6**) generated an [M-H-36]^−^ ion peak at 501, an [M-H-52]^−^ ion at 485, an [M-H-138]^−^ ion at 399, an [M-H-202]^−^ ion at 335 and an [M-H-287]^−^ ion at 250 in its mass spectrum, indicating neutral loss of H_2_O, C_4_H_4_, C_7_H_6_O_3_, C_11_H_6_O_4_ and C_16_H_15_O_5_ moieties, respectively, from the precursor ion. The observed mass of the compound was 538.09 mDa with a mass error of 1.9 mDa. Based on the mass fragmentation pattern and reported literature, compound (**6**) might be robustaflavone [43,44]. The mass spectrum of metabolite (**7**) presented an [M-H]^−^ ion peak at m/z 611 with the molecular formula C_27_H_32_O_16_, and was in agreement with data reported in the literature [45]. The peaks at 431 [M-H-180]^−^, 269 [M-H-342]^−^ and 163 [M-H-448]^−^ suggested the possible loss of C_6_H_12_O_6_, C_14_H_14_O_10_ and C_21_H_20_O_11_, respectively, from the precursor ion at 611. Metabolite (**7**) was, therefore, identified tentatively as hydroxysafflor yellow A. The MS/MS spectrum of metabolite (**8**) displayed a deprotonated [M-H]^−^ ion peak at 353 with a predicted molecular formula C_16_H_18_O_9_, which agrees with literature data [46]. The ion peaks at 233 [M-H-120]^−^ and 205 [M-H-148]^−^ may be due to removal of C_4_H_8_O_4_ and C_5_H_8_O_5_, respectively, from the precursor ion at 353. Metabolite (**8**) was probably undulatoside A with an observed mass of 354.09. The observed mass for compound (**9**) was 756.21 mDa with a predicted formula of C_34_H_44_O_19_. The fragment information at 723 [M-H-32]^−^, 635 [M-H-120]^−^ and 295 [M-H-460]^−^ could be explained by possible loss of structural moieties like CH_3_OH, C_4_H_8_O_4_ and C_23_H_24_O_10_, respectively, from the precursor ion. Examination of a mass data bank (PubChem) suggested compound (**9**) was 3-O-[β-D-glucopyranosyl-(1–2)]-β-D-glucopyranosyl-7-O-α-L-glucopyranosyl-kaempferol. Compound (**10**) with a molecular formula C_33_H_40_O_14_ appeared with an observed mass of 660.23 mDa, having mass error of −2.3 mDa.

Furthermore, the MS/MS spectrum of compound (**10**) showed fragment ions at 599 [M-H-60]^−^, 497 [M-H-162]^−^, 479 [M-H-180]^−^ and 395 [M-H-264]^−^, indicating possible loss of C_2_H_4_O_2_, C_6_H_10_O_5_, C_6_H_12_O_6_ and C_11_H_20_O_7_ groups, respectively, from the precursor ion, which agrees with previous literature. Compound (**10**) was identified tentatively as 2″-O-Rhamno-sylicariside [47]. Compound (**11**), with a predicted molecular formula of C_30_H_22_O_9_ and observed mass of 526.12 mDa, was designated as dendrocandin H [48]. The peaks at 495 [M-H-30]^−^, 479 [M-H-46]^−^, 467 [M-H-58]^−^, 185 [M-H-340]^−^ and 141 [M-H-384]^−^, may indicate possible elimination of CH_2_O, C_2_H_6_O, C_2_H_2_O_2_, C_19_H_16_O_6_ and C_21_H_20_O_7_, respectively, from the precursor ion. The MS/MS spectrum of compound (**12**) provided [M-H-152]^−^ fragments at m/z 425. This possible fragmentation was probably due to Retro Diels Alder rearrangement of the heterocyclic ring. Other fragments at 407 ([M-H-170]^−^), might be due to consequent fragmentation of the heterocyclic ring and neutral loss of H_2_O. The peak at 451 ([M-H-126]^−^) could result from the cleavages between C-C and O-C of one pyran ring, and the peak at m/z 289 ([M-H-288]^−^) might be appeared by the cleavage of an interflavanic bond. Thus, compound (**12**) may be named as procyanidin B2 [49]. Compound (**13**) had a predicted formula of C_18_H_26_O_10_ with an observed mass of 402.15 mDa and a mass error -0.6 mDa. The MS/MS spectrum of the ion at 401 revealed the successive fragment ion peaks at 269 [M-H-132]^−^, 161 [M-H-240]^−^ and 101 [M-H-300]^−^ due to possible cleavage of C_5_H_8_O_4_, C_12_H_16_O_5_ and C_14_H_20_O_7_. Hence, compound (**13**) was tentatively identified as benzyl alcohol xylopyranosyl (1–6) glucopyranoside [48,50]. The characteristic fragment ions for compound (**14**) in the MS/MS spectrum were at 401 [M-H-46]^−^, 269 [M-H-178]^−^, 161 [M-H-286]^−^ and 101 [M-H-346]^−^ due possible loss of C_2_H_6_O, C_6_H_10_O_6_, C_13_H_18_O_7_ and C_15_H_22_O_9_, respectively, from the ion at 447. It was, therefore, identified tentatively as 8-O-acetyl shanzhiside methyl ester [51,52]. Compound (**15**) exhibited a characteristic fragmentation pattern in its MS/MS spectrum. The daughter ions were at 635 [M-H-162]^−^, 577 [M-H-220]^−^ and 487 [M-H-310]^−^, which were attributed to the probable elimination C_6_H_10_O_5_, C_9_H_16_O_6_ and C_14_H_14_O_8_ groups, respectively, from the precursor ion. Based upon the mass fragmentation pattern and reported literature, compound (**15**) was characterized as rindoside [53]. Compound (**16**) generated peaks at 245 [M-H-44]^−^, 203 [M-H-86]^−^ (cleavage of flavan-3-ol ring), 161 [M-H-128]^−^ (as base peak) and 123 [M-H-166]^−^, attributed to cleavage of C_2_H_4_O, C_4_H_6_O_2_, C_6_H_8_O_3_ and C_9_H_10_O_3_. Compound (**16**) was identified tentatively as epicatechin and was in accordance with literature data [54,55]. Compound (**17**) was identified as sesamol, with a molecular formula of C_7_H_6_O_3_. A daughter ion produced at 121 suggested the elimination of an -OH group, in accordance with the literature data [56]. Compound (**18**) corresponds to the predicted molecular formula of C_34_H_58_O_6_ with an observed mass of 562.42 mDa [56,57]. In its mass spectrum, the fragments were at 543 [M-H-18]^−^, 435 [M-H-126]^−^, 407 [M-H-154]^−^, 381 [M-H-180]^−^, 329 [M-H-232]^−^, 311 [M-H-250]^−^ and 245 [M-H-316]^−^, which might be attributed to the loss of H_2_O, C_9_H_18_, C_11_H_22_, C_6_H_12_O_6_, C_10_H_16_O_6_, C_11_H_22_O_6_ and C_23_H_40_ moieties from the parent. Compound (**18**) was, therefore, tentatively identified as campesterol-β-D-glucoside. Compound **(19)** was identified as apocynoside I. According to mass spectral information, compound **(19)** gave product ion peaks at 205 [M-H-180]^−^ and 153 [M-H-232]^−^. This might be due to cleavage of C_6_H_12_O_6_ and C_10_H_16_O_6_ residues obtained from the precursor ion, respectively [58]. Compound (**20**) was detected with a predicted molecular formula of C_45_H_38_O_18_. Fragment ion peaks at 695 [M-H-170]^−^, 577 [M-H-288]^−^, 559 [M-H-306]^−^ and 287 [M-H-578]^−^ might result from cleavage of C_8_H_10_O_4_, C_15_H_12_O_6_, C_15_H_14_O_7_ and C_30_H_26_O_12_ moieties from the parent ion. Thus, the tentative identification of compound (**20**) was arecatannin A 1. The mass spectrum of metabolite (**21**), with molecular formula C_15_H_12_O_4_, showed fragment ions at 229 [M-H-26]^−^, 187 [M-H-68]^−^, 171 [M-H-84]^−^ and 97 [M-H-158]^−^, which could be explained by the elimination of C_2_H_2_, C_4_H_4_O, C_5_H_8_O and C_10_H_6_O_2_, respectively, from the precursor ion. The structure of this compound was identified tentatively as furoaloesone. Compound (**22**), with the formula C_30_H_24_O_9_, produced ions at 419 [M-H-108]^−^, 301 [M-H-226]^−^, 273 [M-H-254]^−^ and 165 [M-H-362]^−^, respectively, from the precursor ion due to the possible loss of C_7_H_8_O, C_12_H_18_O_4_, C_15_H_10_O_4_ and C_21_H_14_O_6_, respectively. Comparing the data with reported literature, compound (**22**) was characterized as mahuannin E [59]. In its MS/MS spectrum, compound (**23**) (C_27_H_30_O_16_) produced daughter ions (typical for rutin) at 489 [M-H-120]^−^ (as a result of possible loss of a rhamnose moiety) and at 301 [M-H-308]^−^ (formed after direct loss of rutinoside residue). Another fragment at 109 [M-H-500]^−^ might have appeared by breakage of a C_21_H_24_O_14_ moiety from the precursor ion. Therefore, these data, and comparison with reported literature, led to characterization/identification of metabolite (**23**) as rutin [60,61].

### 2.6. Docking Studies

**Figure 6:**
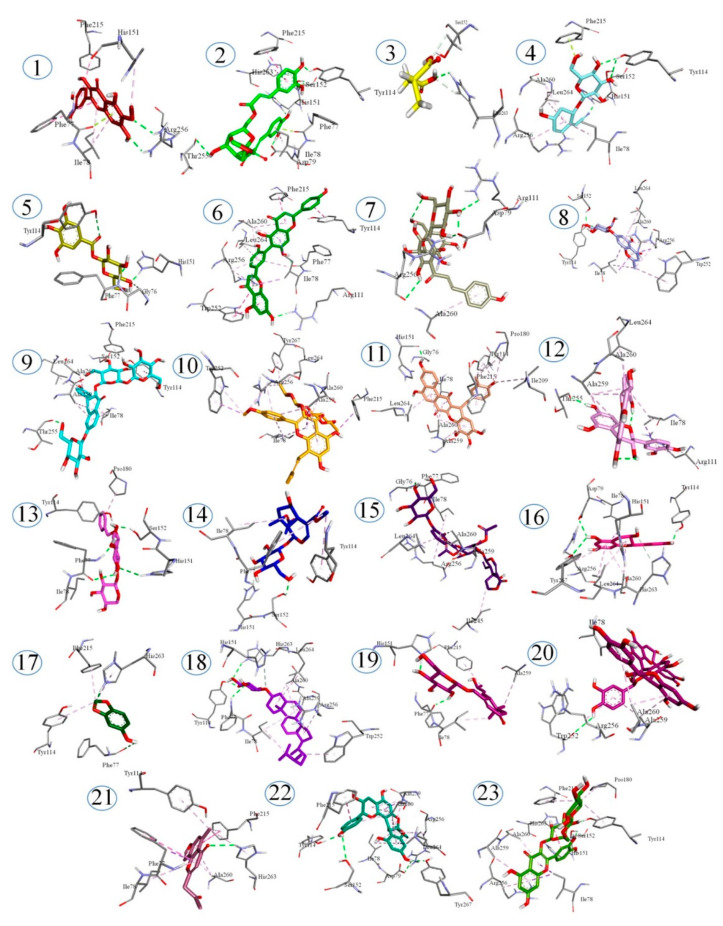
The three-dimensional (3-D) interaction plots of identified phytocompounds (1. Patuletin, 2. Erigoster A, 3. Methyl succinic acid, 4. Menisdaurin, 5. 1-Galloyl-β-D-glucose, 6. Robustaflavone, 7. Hydroxysafflor yellow A, 8. Undulatoside A, 9. 3-O-[β-D-Glucopyranosyl-(1→2)]-β-D-glucopyranosyl-7-O-α-L-glucopyranosylkaempferol, 10. 2″-O-Rhamno-sylicariside II, 11. Dendrocandin H, 12. Procyanidin B2, 13. Benzyl alcohol xylopyranosyl (1→6) glucopyranoside, 14. 8-O-Acetyl shanzhiside methyl ester, 15. Rindoside, 16. Epicatechin, 17. Sesamol, 18. Campesterol-β-D-glucoside, 19. Apocynoside I, 20. Arecatannin A1, 21. Furoaloesone, 22. Mahuannin E and 23. Rutin) into the active site regions of the PL enzyme.

The affinity of identified phytochemicals with PL was predicted by the docking studies. Bonding energies of the phytochemicals with lipase (PDB No. 1LPB), calculated by the MOE are given in Table 3. Binding energies calculated for compounds ranged from −15.0210 to −10.0161 KJ/mol. The lowest energy was exhibited by Undulatoside A. All the phytochemicals identified in the *C. fistula* extract showed binding energies lower than orlistat, which was found to be −9.1309 kJ/mol for PL. The binding profiles of the phytochemicals are presented in the Figure 6. The binding profile of orlistat reveals that it interacts with TYR114 and PHE77 residues by hydrogen bonds, while it can interact with the ILE78, TYR114, SER152, PRO180, PHE215, ARG256, ALA259, ALA260, LEU264 residues through other nonbonding interactions. The catalytic tirade of 1LPB consists of SER152, HIS263 and ASP176, but SER152 is mainly responsible for the activity of lipase. So, the compounds showing interactions with these amino acid residues can alter the lipase activity. However, other amino acid residues like PHE77 and TYR114 are also key residues of the active pocket of lipase (PDB No. 1LPB). The compounds, showing the lowest binding energies, exhibited interactions with the key residues of lipase. The binding profile of undulatoside A shows that it can interact with SER152, TYR114 through a hydrogen bond, and shows pi-sigma interaction with ILE78 and ARG256, while it interacts with ALA260, LEU264 and TRP252 by pi-alkyl interaction. Conventional hydrogen bonding with SER152 was also depicted by the bindings profiles of erigoster A, menisdaurin, 3-Feruloyl-4-caffeoylquinic acid, rutin, mahuannin E, 8-O-acetylshanzhiside methyl ester, benzyl alcohol xylopyranosyl(1–6)glucopyranoside and 3-O-[β-D-Glucopyranosyl-(1–2)]-β-D-glucopy-ranosyl-7-O-α-L-glucopyranosylkaempferol. From the low binding energies and strong hydrogen bond interactions with key amino acid residues of the active sites of lipase, it can be predicted that *C. fistula* extract can help to reduce lipolytic activity in a considerable manner, hence controlling obesity.

### 2.7. Computational Studies

Plants are considered as essential source of phytocompounds. Many therapeutic agents have been identified in medicinal plants. However, some of these medicinal plants might contain a wide variety of chemical compounds, often with undetermined biological properties [62]. The antioxidant activities of polyphenolic compounds depend upon their ability to resist harmful effects of free radical species. There exists a close relationship between structural properties and antioxidant activities. Among various structural features, the effect of substituent is the most significant factor that influences antioxidant activities of polyphenolic compounds [63]. A quantum computational approach has proven useful in determining the structure, reaction and the electronic structures of the molecules [64]. It is a cost-effective and predictive method. It provides valuable information about the antioxidant character of polyphenolic phytocompounds [65]. The density functional descriptors for structure-activity relationships are pivotal in the examination of interactional aspects, active sites, and possible biological functions of phytoconstituents. It is noteworthy to shed light on FMOs, IP, EA and MEP to explore the unique and important biological and pharmacological properties of compounds. The results acquired from the computational study of the identified phytoconstituents, in addition to molecular docking studies, might be very helpful in exploring their possible phytochemical role. Hence, these findings may be helpful for future studies related to isolation and characterization of potent metabolites for the pharmacological/nutraceutical applications.

**Figure 7:**
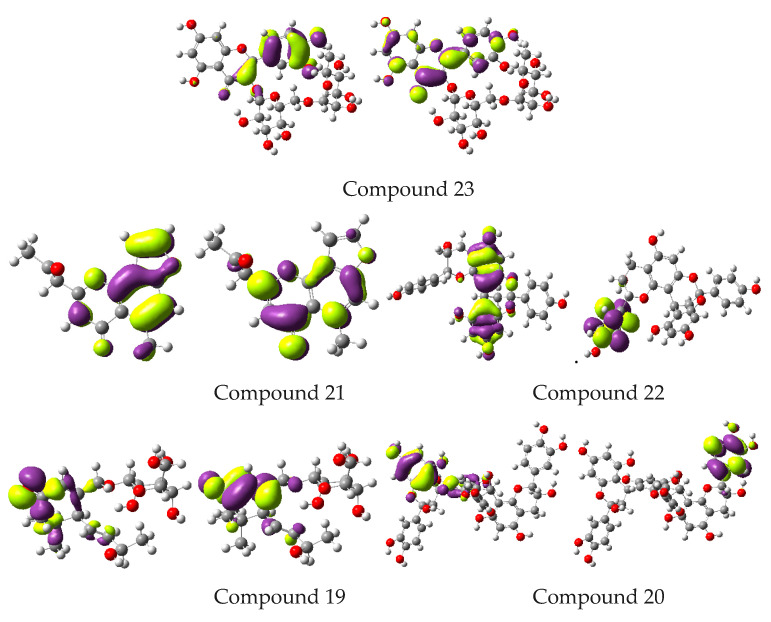

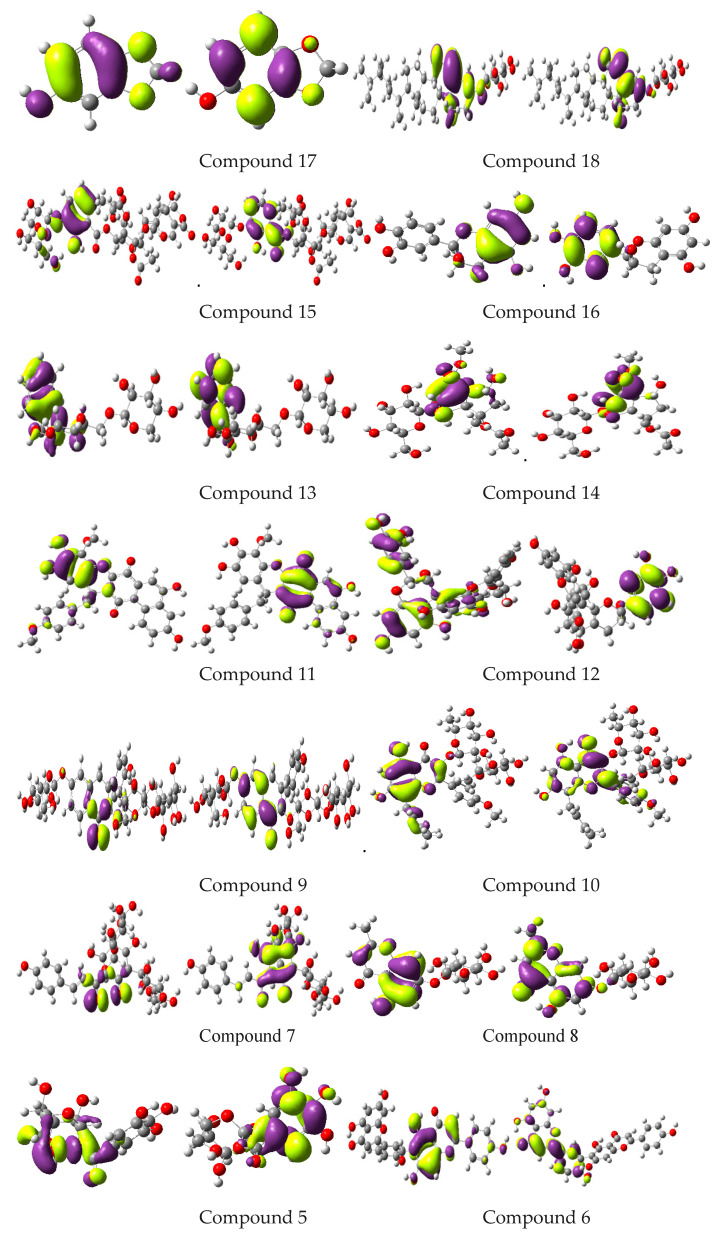

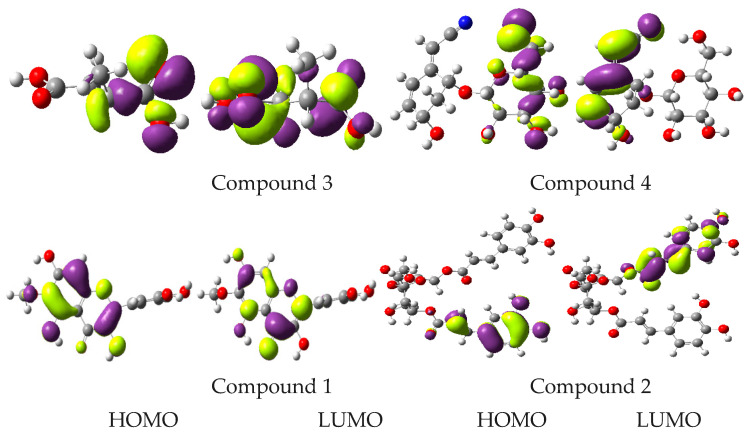
Spatial distribution of frontier molecular orbitals at the ground state (Contour value = 0.035).

The current work involved the computation of active sites for the most relevant and practical information. The electronic environments of functional groups of compounds present in a particular extract, and interaction with active sites of enzymes, provides a great deal of information about medicinal potential. The information on electronic properties and MEP exploration is significant in elaborating the role of active sites in compounds displaying fascinating properties. The charge density/spatial distribution of FMOs, i.e., the highest occupied molecular orbitals (HOMOs) as well as lowest unoccupied molecular orbitals (LUMOs) is illustrated as Figure 7.

Spatial distribution exposed detailed intramolecular charge transfer from HOMOs to LUMOs. The antioxidant and free radical scavenging activity of bioactive molecules and entities are interlinked with the charge density distribution of FMOs, which illuminate the possible active sites. The spatial distribution of FMOs overlapping molecules highlighted that these phytomolecules could be of great therapeutic use regarding their antioxidant and antiobesity properties. Energies of FMOs and HOMO-LUMO energy gaps (*E_gaps_*) are significant parameters to understand the electronic nature of biologically active substances. Energies of HOMOs (*E_HOMOs_, E_HOMOs-1_*), LUMOs (*E_LUMOs_, E_LUMOs+1_*) and *E_gaps_* of different phytocompounds at B3LYP/6-31G** level are shown in Table 4.

**Figure 8:**
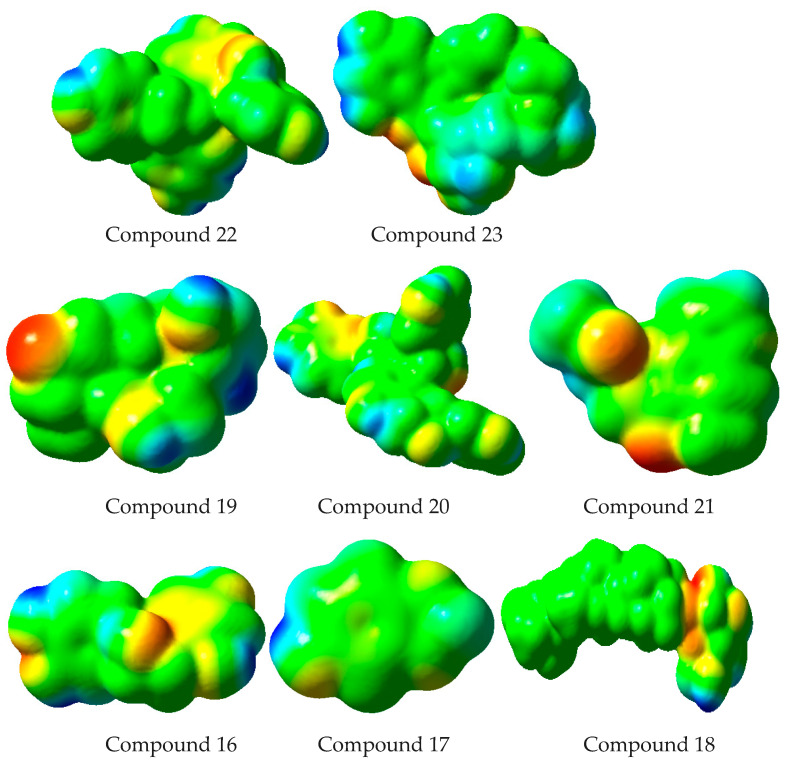

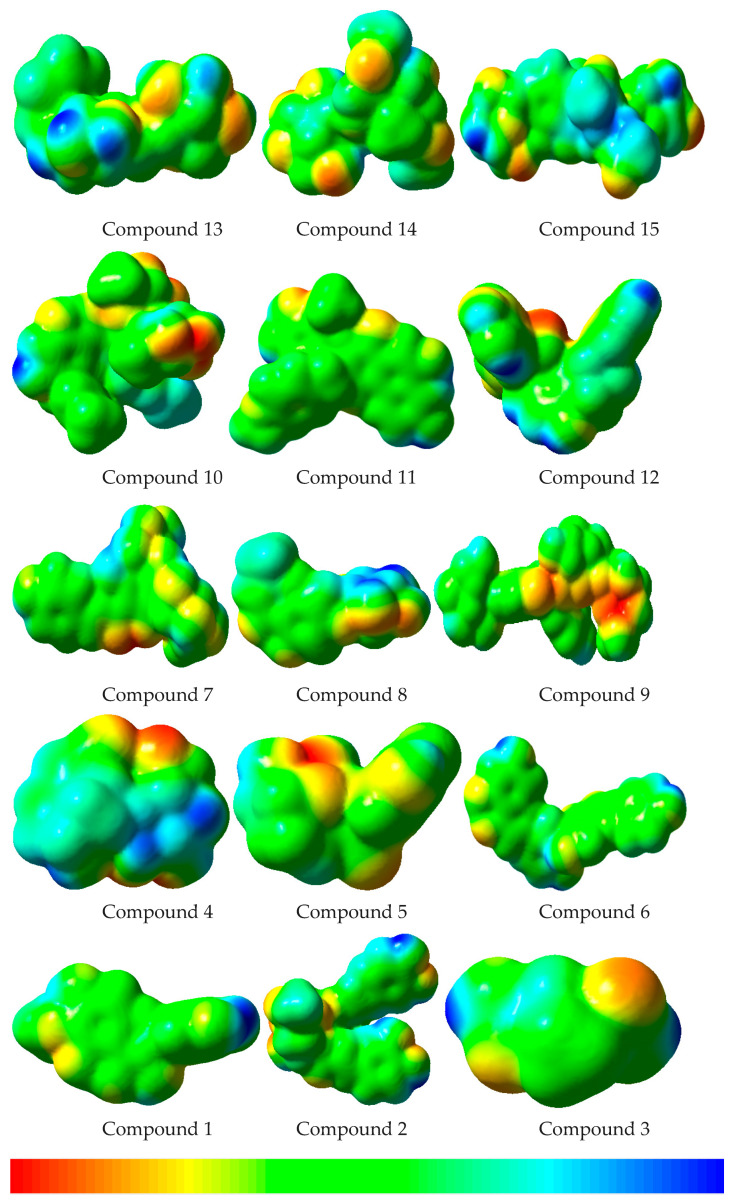
Molecular electrostatic potential surfaces views of studied compounds.

Studies have shown that diffraction approaches can be very helpful to determine MEP on practical grounds [66]. The MEP illustrates a broad range of nuclear and electronic charge distributions, and helps in understanding and predicting the reactivity of different entities [67]. In Figure 8, the MEP mapped for identified phytocompounds is shown in color schemes. Higher negative potential regions (suitable for electrophilic attack) are indicated by the red color, and higher positive potential regions (favoring nucleophilic attack) are specified by the blue color. The declining order of MEP is blue > green > yellow > orange > red. Here, the red color indicates the strongest repulsion while the blue color indicates the phase of substantial attraction. Figure 8 revealed that oxygen atoms of the methoxy, hydroxy, keto or carboxylic group, as well as -CN, would have negative electrostatic potential. On the other hand, hydrogen atoms present in hydroxyl and methyl groups have positive electrostatic potential. These results clearly indicate that in the case of electrophilic or nucleophilic attack, reasonable repulsion would be at oxygen, CN and -COOH/-H, while significant attraction would be at –H/oxygen atoms, CN, and -COOH groups, respectively.

Antioxidants have the ability to donate electrons to free radicals and stabilize the radical. The antioxidant ability of a compound can be determined by IP in a single electron transfer mechanism. The IP is a vital parameter that illuminates electron transfer range (computed as IP = *-E_HOMO_*). Compounds having low IP values can reflect higher antioxidant activity due to ease of electron transfer. In the current study, phenol was used as standard, and the findings revealed that the understudy phytochemicals exhibited low IP values when compared to phenol i.e., 8.33 eV [68], which would be a supporting factor for antioxidant potential. The data of IP predicted that the isolated compounds would have good antioxidant activities. It can be interpreted from IP values that the compounds may show a better electron donating effect in comparison to phenol with maximum *E_HOMO_* and minimum IP values. The MEP mapping of active sites, spatial distribution of FMOs and IP indicated that these phytochemicals would be reactive with good antioxidant and antiobesity capability.

The in vitro antioxidant activity and PL inhibition of *C. fistula* extracts were supported by the sound evidence of computational data. The secondary metabolites identified in hydroethanolic extract of *C. fistula* were mainly responsible for the antioxidant and PL inhibitory action. The experimental outcomes suggest that the *C. fistula* has medicinal potential related to oxidative stress and obesity. Therefore the *C. fistula* may be a potential candidate for food fortification to add antiobesity functionalities in food systems.

## 3. Materials and Methods

### 3.1. Plant Collection and Identification

Mature leaves of *C. fistula* were collected from Azad Jammu & Kashmir, Pakistan and identified at the Department of Botany University of Gujrat, Gujrat Pakistan. For identification, a voucher specimen # UOGCHEM46/2018 was also deposited.

### 3.2. Extract Preparation

The freshly collected leaves were cleaned, dried, and quenched in liquid nitrogen immediately. The purpose of quenching was to preserve secondary metabolites for longer periods. After that, lyophilization was carried out at −68 °C for 48 h. The lyophilized leafy material was converted into a fine powder (60-mesh size) and kept at extremely low temperature (−80 °C) for further analysis. Dried powder (10 g) was then extracted with 100 mL of hydroethanolic solvent systems of various concentrations (i.e., 20–80% hydroethanol (*v*/*v*), and pure ethanol) at 35 ± 0.2 °C and a humidity of 25 ± 5% for a period of two days. Sample extracts under study were then vortexed for 2 h utilizing Wise-Mix SHO1D, DAIHAN Scientific, Korea, ultrasonicated for 30 min at 20 KHz. After centrifugation for 10 min at 13,000 rpm, filtration was carried out by applying a filtration assembly connected with a vacuum pump, the filtrate evaporated with the help of rotary vacuum evaporator to avoid the loss of phytoconstituents and freeze dried again at −68 °C for further use.

### 3.3. Phytochemical Screening

#### 3.3.1. Total Phenolic Contents

The TPCs of the extracts were determined by Folin-Ciocalteu’s (FC) method with slight modifications [69]. Briefly, the sample extracts were dissolved in methanol and mixed with FC-reagent. After that, samples were mixed with 20% sodium carbonate solution (4 mL) and incubated for 90 min at room temperature. The absorbance was noted at 750 nm. Gallic acid was employed as a reference and results were presented as milligram of gallic acid equivalent per gram (mg GAE/g).

#### 3.3.2. Total Flavonoid Contents

The TFCs of each extract were investigated by a reported spectrophotometric method with slight changes [70]. Briefly, sample extracts were dissolved in methanol followed by adding 0.10 mL sodium nitrite (0.5 *M*), 0.15 mL aluminum chloride of (0.3 *M*) solution and 3.4 mL of 30% methanol. After a short while, 1 mL sodium hydroxide (1 *M*) was added, and measurement of absorbance were performed at 510 nm. Rutin was employed as a reference and results were reported as milligram of rutin equivalent per gram (mg RE/g).

### 3.4. Antioxidant Activity

The DPPH (2,2-diphenyl-1-picrilhydrazyl) scavenging capability assay was applied to appraise antioxidant effect of each extract. Analysis was conducted as per a previously established scheme with slight modifications [25]. Briefly, the sample extracts were mixed with the solution of DPPH in methanol and after incubation for 20 min at 35 °C absorbance was recorded at 517 nm. The free radical scavenging ability (%) was computed as follows.

$$\mathrm{Scavenging}\;\%=\left(\frac{\mathrm{Absorbance}\;\mathrm{of}\;\mathrm{control}-\mathrm{Absorbance}\;\mathrm{of}\;\mathrm{blank}}{\mathrm{Absorbance}\;\mathrm{of}\;\mathrm{control}}\right)$$

Butylatedhydroxyanisole (BHA) was employed as reference standard and outcomes were reported as IC_50_ (µg/mL). All the experiments were performed thrice.

### 3.5. Pancreatic Lipase Inhibitory Assay

The capability of optimized extracts to inhibit the activity of PL was appraised using a previously described method [3] with minor changes. In brief, sample extracts were prepared in Tris-HCl buffer (0.01 *M*) followed by addition of Arabic gum (10 g) and olive oil (10% *w*/*v* in Tris-HCl buffer) mixture utilizing a homogenizer for 20 high pressure homogenization passes. Extracts were resuspended in methanol, allowed to react with a solution of 1mg/mL PL (25 units/mL), and 2 mL of emulsion were used for assay. The sample extracts were then incubated at 4 °C for 30 min followed by adding substrate solution (2 mL) and reincubated at 37 °C for 30 min. After that, the reactions were stopped by adding a mixture acetone and ethanol (1:1) into the reaction mixtures and titrated against a solution of sodium hydroxide (0.02 *M*) for the neutralization of free fatty acids (pH, 9.4). The PL inhibitory activity (%) was computed as follows.

Inhibition (%) = 100% − [(V_s_/V_c_) × 100]

where, V_c_ = Volume of base utilized for control and vs. = Volume of base utilized for sample

### 3.6. UHPLC-QTOF-MS/MS-Based Metabolite Profiling

UHPLC-QTOF-MS/MS technique was employed for the identification of metabolites in the most potent extract. The methanolic extract was prepared by dissolving dry extract in aqueous CH_3_OH. The obtained extract was filtered using a poly-tetrafluoroethylene filter (0.45 µm size). UHPLC-based separation was performed on a Waters ACQUITY chromatograph with a binary pump system, a degasser and autosampling assembly. An ACQUITY UPLC HSS T3 column (C-18 with dimensions of 1.8 μm × 2.1 mm x 100 mm) was used at 40 °C. Gradient elution was conducted by means of a binary solvent system (water having formic acid (0.1%) as solvent A and acetonitrile as solvent B) with variable compositions. The composition of the mobile phase changed during experiments in a pattern of 1% B for 0 min; 1% B for 0.5 min; 35% B for 16 min, 100% B for 18 min; 1% B for20 min with flow rate of 0.6 mL/min. A sample volume of 1 μL was injected. The chromatograph was equipped with a hybrid mass spectrometer by Waters (Vion IMS QTOF). Ionization was done in both negative and positive electrospray ionization mode. An operational capillary voltage of 1.50 kV was used, and a reference capillary voltage of 3.00 kV was set for experimental runs. A source temperature of 120 °C, desolvation gas flow of 800 L/h and desolvation gas temperature of 550 °C was used. A cone gas flow of 50 L/h was established using Nitrogen (>99.5%). Data were obtained in high definition MS^E^ mode ranging from 50 to 1500 m/z at 0.1 s/scan. Argon gas (99.999%) was utilized as the collision-induced dissociation gas. A mass spectral data base compound identification was utilized.

### 3.7. Molecular Docking Studies

Docking studies of all identified phytocompounds were conducted by Molecular Operating Environment (MOE 2016.08). The 3-D structure of PL was downloaded and phytocompounds were docked into its active sites. Binding energies were computed after the preparation of ligand-enzyme conformations and energy minimization. Both hydrophilic as well as hydrophobic interactions on different amino acid residues were studied. Results of molecular docking were assessed and surfaces were analyzed graphically applying MOE as well as discovery-studio-visualizer.

### 3.8. Computational Studies

Computational approaches provide significant information regarding free radical scavenging activity of antioxidants. The computational protocol known as quantum chemical-based test can be utilized for quantification of antioxidant protection for overall radical scavenging activity and to predict trends [71]. The specialty of computational methods in predicting antioxidant properties of phytocompounds is that ionization energy values can be obtained for compounds under study [72]. Presently, first-principles calculations are of great significance to explain the different characteristics of materials. Numerous investigations have revealed the efficacy and utility of density functional theory (DFT) as a useful analytical tool to determine the electronic environment to correlate with the actual data [73,74,75]. DFT is known for optimization of ground-state geometries of molecules belonging to various classes [76]. B3LYP has been established as a rational approach among different DFT functionalities [77]. The current study involved the optimization of S_0_ geometry by B3LYP/6-31G** level in Gaussian16 software.

### 3.9. Statistical Analysis

Results were summarized in the form of mean (±) standard error of experiments carried out in triplicate. Differences among data were executed by one way analysis of variance (ANOVA) utilizing Minitab (version-17) statistical software.

## 4. Conclusions

The results confirmed strong antioxidant and antiobesity attributes of *C. fistula* leaves. The 80% hydroethanolic leaf extract of *C. fistula* was established as the better fraction to exhibit antioxidant activities and PL enzyme inhibition. Metabolite profiling of same extract explored the presence of 23 bioactive constituents, and many of them were pharmacologically effective. The findings of molecular docking and computational studies explored possible effects of the identified phytocompounds as antioxidant and antiobesity agents. Hence, the 80% hydroethanolic leaf extract of *C. fistula* with promising antioxidant and lipase inhibitory activity can be used for innovative naturopathic antiobesity approaches. The considerable antioxidant activity, metabolite availability and antiobesity attributes identify *C. fistula* as a reasonable indigenous candidate for food fortification. The extract may be further processed for development of antiobesity functional foods and phytopharmaceuticals.

## Figures and Tables

**Table 1:** TPCs and TFCs of different extracts from *C. fistula*.

Extract Type	TPCs (mg GAE/g Extract)	TFCs (mg RE/g Extract)
20% hydroethanolic	152.19 ± 1.4 ^b^	78.4 ± 2 ^b^
40% hydroethanolic	181.2 ± 1.9 ^c^	99.8 ± 1.18 ^c^
60% hydroethanolic	190.6 ± 30 ^d^	102.6 ± 1.0 ^c^
80% hydroethanolic	201.3 ± 2.6 ^e^	116.3 ± 2.4 ^d^
Pure ethanolic	138.5± 2.3 ^a^	66 ± 1.7 ^a^

Letters ^(a–e)^ indicate statistically significant numbers with *p* < 0.05. The values that do not share a letter are statistically significant.

**Table 2:** Mass spectral information of the compounds identified from the 80%hydroethanolicleaf extract of *C. fistula* by UHPLC-QTOF-MS/MS analysis.

Sr. #	Observed Mass (mDa)	Predicted Formula	Mass Error (mDa)	Matched Identity	MS/MS Fragments (m/z)
1	332.05	C_16_H_12_O_8_	−0.7	Patuletin	317,300,164
2	558.14	C_27_H_26_O_13_	2.7	Erigoster A	517,437,113
3	132.04	C_5_H_8_O_4_	−0.3	Methyl succinic acid	101, 85,71,59
4	313.11	C_14_H_19_NO_7_	−1.5	Menisdaurin	277,211,174,113
5	332.07	C_13_H_16_O_10_	−0.1	1-Galloyl-β-D-glucose	168
6	538.09	C_30_H_18_O_10_	1.9	Robustaflavone	501,485, 399, 335,250
7	612.16	C_27_H_32_O_16_	0.7	Hydroxysafflor yellow A	611,431, 269, 163
8	354.09	C_16_H_18_O_9_	−0.1	Undulatoside A	233,205
9	756.21	C_34_H_44_O_19_	1.1	3-O-[β-D-Glucopyranosyl-(1→2)]-β-D-glucopyranosyl-7-O-α-L-glucopyranosyl-kaempferol	723, 635,617,419,295
10	660.23	C_33_H_40_O_14_	−2.3	2″-O-Rhamno-sylicariside II	637,599,497,479,395
11	526.12	C_30_H_22_O_9_	2.2	Dendrocandin H	495,479,467,185,141
12	578.14	C_30_H_26_O_12_	−0.1	Procyanidin B2	451,425,407,311,289,245,161
13	402.15	C_18_H_26_O_10_	−0.6	Benzyl alcohol xylopyranosyl(1→6)glucopyranoside	343,269,161,101
14	448.15	C_19_H_28_O_12_	−0.4	8-O-Acetyl shanzhiside methyl ester	437,401,269,161,101
15	798.22	C_35_H_42_O_21_	0.7	Rindoside	635,577,487,446
16	290.07	C_15_H_14_O_6_	−0.6	Epicatechin	271,245,203,161,123
17	138.03	C_7_H_6_O_3_	−0.5	Sesamol	125,121
18	562.42	C_34_H_58_O_6_	2.5	Campesterol-β-D-glucoside	543,435,407,381,311,289,245,203,164
19	386.19	C_19_H_30_O_8_	−0.6	Apocynoside I	367, 205,153
20	866.20	C_45_H_38_O_18_	3.8	Arecatannin A1	748,695,577,559,405,287
21	256.07	C_15_H_12_O_4_	−0.5	Furoaloesone	229,187,171,97
22	528.14	C_30_H_24_O_9_	1.4	Mahuannin E	419,301,273,255,165
23	610.15	C_27_H_30_O_16_	0.3	Rutin	545,489,301,243,109

**Table 3:** Binding energy data for identified phytocompounds docked against the PL enzyme.

Sr. #	Compound Name	Scores (S-value)	Hydrogen Bonding	Other Nonbonding Interactions
1	Patuletin	−14.2007	ARG 256	PHE77, PHE215, ILE78, HIS151
2	Erigoster A	−14.5517	SER152, TYR114, THR255, ASP78, HIS151	PHE77, ILE78, PHE215, HIS263
3	Methyl succinic acid	−10.2645	HIS263	SER152
4	Menisdaurin	−13.3161	SER152, TYR114, HIS151	PHE77, ILE78, ALA260, ARG26, LEU264
5	1-Galloyl-β-D-glucose	−14.3628	TYR114, PHE77, HIS151	GLY76
6	Robustaflavone	−12.5894	PHE77, ARG 256, ARG111	TRP252, ILE78, ALA260, TYR114, PHE215, LEU264
7	Hydroxysafflor yellow A	−14.6837	ARG256, ASP79, ARG111	ALA260
8	Undulatoside A	−15.0210	SER152, TYR114	ILE78, ALA260, LEU264, ARG256, TRP252
9	3-O-[β-D-Glucopyranosyl-(1→2)]-β-D-glucopyranosyl-7-O-α-L-glucopyranosylkaempferol	−12.0339	SER152, TYR114, THR255	PHE215, ILE78, LEU265, ALA260, ALA259
10	2″-O-Rhamno-sylicariside II	−13.5572		PHE215, LEU264, TYR267, ALA260, ALA259, ILE78, ARG256, TRP252
11	Dendrocandin H	−14.3445	HIS151	ILE78, LEU264, GLY76, TYR114, PHE215, PRO180, ILE209, ALA259, ALA260
12	Procyanidin B2	−12.3625	THR255	ALA260, LEU264, ARG11, ALA259, ILE78
13	Benzyl alcohol xylopyranosyl (1→6) glucopyranoside	−13.9147	SER152, PHE77, TYR114, HIS151	PRO180
14	8-O-Acetyl shanzhiside methyl ester	−12.9175	SER152	TYR114, ILE78, PHE77, HIS151
15	Rindoside	−10.0161	PHE77	LEU264, ILE78, ARG260, ARG256, ALA259, ILE245
16	Epicatechin	−13.6194	ASP79, TYR114, TYR267	HIS263, LEU264, ARG256, ALA260, HIS151, ILE78
17	Sesamol	−10.5688	PHE77, HIS263	PHE215, TYR114
18	Campesterol-β-D-glucoside	−11.5302	PHE77, TYR114, HIS263	ALA259, ALA260, ARG256, ILE78, LEU264, TRP252
19	Apocynoside I	−12.3624	PHE77, HIS151	ALA259, ILE78, PHE215
20	Arecatannin A1	−11.4091	TRP252	ILE78, ARG256, ALA259, ALA260
21	Furoaloesone	−11.0088	HIS263	PHE77, ALA260, PHE215, TYR114, ILE78
22	Mahuannin E	−13.4295	SER152, TYR114, ASP79, TYR267	ILE78, ALA259, PHE215, ALA260, LEU264, ARG356
23	Rutin	−14.2859	SER152, HIS151, ARG256, TYR114	HIS263, ALA260, ILE78, ALA259, PRO180, PHE215
24	Orlistat	−9.1309	TYR114, PHE77	ILE78, TYR114, SER152, PRO180, PHE215, ARG256, ALA259, ALA260, LEU264

**Table 4:** Ground state HOMO energies (*E_HOMO_*), LUMO energies (*E_LUMO_*), HOMO-LUMO-*E_gap_*, EA and IP in eV of identified phytocompounds.

Compounds	*E_HOMO_*	*E_LUMO_*	*E_gap_*	EA	IP
1	−5.77	−1.41	4.36	1.41	5.77
2	−5.79	−1.68	4.11	1.68	5.79
3	−7.45	−0.22	7.67	0.22	7.45
4	−6.55	−2.11	4.44	2.11	6.55
5	−3.74	−0.04	3.74	0.04	3.74
6	−5.95	−1.58	4.37	1.58	5.95
7	−4.84	−2.63	2.21	2.63	4.84
8	−6.20	−1.44	4.76	1.44	6.20
9	−5.71	−0.84	4.87	0.84	5.71
10	−5.88	−1.50	4.38	1.50	5.88
11	−5.42	−2.95	2.47	2.95	5.42
12	−5.31	−0.04	5.27	0.04	5.31
13	−6.06	−0.32	5.74	0.32	6.06
14	−6.66	−0.96	5.70	0.96	6.66
15	−6.37	−1.90	4.47	1.90	6.37
16	−5.52	−0.15	5.37	0.15	5.52
17	−5.26	0.06	5.32	−0.06	5.26
18	−6.07	0.84	6.91	−0.84	6.07
19	−6.18	−1.21	4.97	1.21	6.18
20	−5.10	−0.17	4.93	0.17	5.10
21	−5.90	−1.15	4.74	1.15	5.90
22	−5.30	−0.37	4.92	0.37	5.30
23	−5.57	−1.57	5.00	1.57	5.57
Phenol	−5.96	0.036	5.99	−0.036	8.11

## Data Availability

Not applicable.
